# Beneficial effect of ER stress preconditioning in protection against FFA-induced adipocyte inflammation via XBP1 in 3T3-L1 adipocytes

**DOI:** 10.1007/s11010-019-03627-3

**Published:** 2019-10-16

**Authors:** Min Wang, Xi Chen, Zhenda Zheng, Shujie Yu, Bin Zhou, Yong Liu, Dinghui Liu, Yanming Chen, Xiaoxian Qian

**Affiliations:** 1 Department of Cardiology, The Third Affiliated Hospital of Sun Yat-sen University, Sun Yat-sen University, 600 Tianhe, Guangzhou, 510630 Guangdong China; 2 Department of Rehabilitation Medicine, The Third Affiliated Hospital, Sun Yat-sen University, Guangzhou, 510630 Guangdong China; 3 Department of Endocrinology, The Third Affiliated Hospital, Sun Yat-sen University, Guangzhou, 510630 Guangdong China; 4 Institute Integrated Traditional Chinese and Western Medicine, Sun Yat-sen University, Guangzhou, 510630 Guangdong China

**Keywords:** Endoplasmic reticulum stress, Inflammation, Protective effects, Free fatty acids, NF-κB

## Abstract

Adipose tissue inflammation is closely associated with the development of obesity and insulin resistance. Free fatty acids (FFAs) are a major inducer of obesity-related insulin resistance. Previously, we reported that endoplasmic reticulum (ER) stress potentially mediated retinal inflammation in diabetic retinopathy. The unfolded protein response (UPR) protects cells against damage induced by oxidative stress. X-box binding protein 1 (XBP1) plays a major role in protecting cells by modulating the UPR. However, the link between ER stress and adipocyte inflammation has been poorly investigated. In the present study, we found that pretreatment of 3T3-L1 adipocytes with a low dose of ER stress inducer tunicamycin inhibited FFA-induced upregulated expression of inflammatory cytokines. In addition, FFAs induced phosphorylation of the p65 subunit of NF-κB was largely inhibited by pretreatment with tunicamycin in 3T3-L1 adipocytes. Knockdown of XBP1 by siRNA markedly mitigated the protective effects of preconditioning against inflammation. Conversely, overexpression of XBP1 alleviated FFA-induced phosphorylation of IκB-α, IKKα/β, and NF-κB, which was accompanied by decreased inflammatory cytokine expression. Collectively, these results imply a beneficial role of ER stress preconditioning in protecting against FFA-induced 3T3-L1 adipocyte inflammation, which is likely mediated through inhibition of the IKK/NF-κB pathway via XBP1.

## Introduction

Obesity is a growing health problem worldwide, which is characterized by adipose cell enlargement and adipocyte inflammation [1–3]. Free fatty acids (FFAs) originating from increased lipolysis of visceral hypertrophic adipocytes lead to the inflammatory status of visceral fat through phosphorylation of several key proteins in the NF-κB signaling pathway [4–6]. Proinflammatory cytokines, such as MCP-1 and IL-6 secreted by inflamed adipose tissue, impair insulin signaling through different mechanisms and result in the development of cardiovascular disease [7], insulin resistance [8], and diabetes [9, 10].

The endoplasmic reticulum (ER) plays principle roles in proper folding and processing of nascent proteins. Perturbed ER functions cause accumulation of misfolded proteins, trigger the apoptotic cascade, and lead to cell apoptosis or death, the process of which is termed ER stress [11, 12]. Recent studies have showed that both ER stress and proinflammatory cytokines are associated with adipose tissue inflammation [13, 14]. Mammalian cells possess a specific signaling pathway known as the unfolded protein response (UPR) [15, 16]. The UPR, which is activated by sublethal stimuli, provides adaptation to subsequent stress and prevents cell death [11]. It consists mainly of three ER stress response transducers: activating transcription factor-6, protein kinase RNA-like endoplasmic reticulum kinase, and inositol-requiring enzyme-1α (IRE1α) [17, 18]. IRE1α homodimerizes and auto-phosphorylates under ER stress, which contributes to increased protein chaperone content, improved ER biogenesis, and an enhanced secretory capacity via X-box binding protein 1 (XBP-1). XBP1 is the only known transcription factor downstream of IRE1α that has previously been demonstrated to initiate the gene transcription, protein folding, and ER-associated protein degradation [19, 20].

A growing body of evidence has shown that preconditioning with ER stress protects various kinds of cell lines against inflammatory and oxidative responses [21–24]. Li et al. [25] reported that preconditioning with ER stress mitigates retinal endothelial inflammation through activation of the XBP-1-mediated UPR. Moreover, Inagi et al. [21] showed that ER stress preconditioning ameliorates mesangioproliferative glomerulonephritis in a rat model. These findings suggest that ER stress may play a protective role against inflammatory response in different cell types.

In our previous study, we found that MCP-1 and IL-6 secretion as well as NF-κB phosphorylation are significantly increased by FFA treatment in 3T3-L1 adipocytes. In the present study, we aimed to elucidate the effects of ER stress preconditioning on FFA-induced inflammatory responses in 3T3-L1 adipocytes and to explore the involved molecular mechanisms. Our data showed that ER stress preconditioning suppressed FFA-induced proinflammatory cytokine secretion and attenuated FFA-induced activation of the NF-κB pathway via XBP1, a principal coordinator of the adaptive UPR. We also elucidated that XBP1 negatively regulated IRE1 phosphorylation and blocked FFA-induced activation of the IRE1/IKK/NF-κB pathway.

## Methods

### Cell culture and adipogenic differentiation of 3T3-L1 preadipocytes

Embryonic mouse fibroblast line 3T3-L1 preadipocytes were purchased from the American Type Culture Collection (Manassas, VA, USA) and cultured in high glucose Dulbecco’s modified Eagle’s medium (DMEM) (Invitrogen, Carlsbad, CA), supplemented with 10% bovine calf serum (Hyclone, Logan, UT) at 37 °C in a atmosphere containing 5% CO2 until differentiation was induced as described previously [26]. Briefly, at 2 days post-confluency, cells were induced by differentiation medium containing 0.5 mM isobutylmethylxanthine, 1 M dexamethasone, 10 µg/ml insulin (MDI; Sigma, St. Louis, MO) for 3 days. Then, the cells were transferred to DMEM with 10 µg/ml insulin and the medium was changed every 2 days. Oil red staining is performed to confirm the maturation of adipocytes.

### Co-culture of adipocytes and macrophages

3T3-L1 adipocytes and RAW264.7 cells were co-cultured using the transwell system as reported previously [27]. Briefly, the 3T3-L1 preadipocytes were cultured and differentiated into 3T3-L1 adipocytes in the lower chamber of the 24-well transwell plate inserts with a 0.4-μm porous membrane (Corning, New York, NY). Then the 3T3-L1 adipocytes with indicated treatments were washed and replaced with DMEM contained 2% FBS. At the same time, the RAW264.7 cells (5.0 × 10^5^ cells/well) were cultured in the upper chamber, and then the two sets were put together by assembling two part of Transwell and incubated in DMEM with 2% FBS at 37 °C for 4 h.

### Cell treatments

Differentiated adipocytes were serum starved for 16 h in DMEM supplemented with 2% FBS before treatment. Tunicamycin was obtained from Sigma-Aldrich. ER stress preconditioning was promoted as described previously [28, 29]. Briefly, the differentiated mature 3T3-L1 adipocytes were pretreated with a very low dose (0.5 µg/ml) of tunicamycin for 4 h followed by treatment with FFA for 4 h. For the macrophage migration assay, the 3T3-L1 adipocytes were preincubated with control siRNA or XBP1siRNA transfection followed by FFA treatment for 4 h in the presence or absence of TM preconditioning, respectively, and then the co-culture systems were incubated in DMEM with 2% FBS at 37°C for 4 h before testing the macrophage migration.

### Macrophage migration assay

The cell migration assay was performed as previously reported [30]. Briefly, non-migrated RAW264.7 cells in the upper chamber were carefully removed and migrated cells on the lower membrane surface were fixed in 1% paraformaldehyde, stained with hematoxylin, and counted (Ten random fields per well). Migrated cell counts were expressed as the mean number of cells per field of view.

### Preparation of fatty acid-albumin complexes

Saturated palmitic acid was used in this study as described previously [6, 31]. Briefly, FFAs were dissolved in ethanol at 200 mmol/l and then combined with 10% FFA-free low endotoxin BSA at concentrations of 1–10 mmol/l. Stock solutions were filter sterilized and stored at − 20 °C. A control solution containing ethanol and BSA were applied as control solution. The final BSA concentration was consistently 1% in all FFA media.

### MCP-1 and IL-6 secretion measurements of by ELISA

The cell culture supernatant was collected after treatments. MCP-1 and IL-6 in supernatants were quantified in triplicate by mouse ELISA kits (R&D Systems, Wiesbaden-Nordenstadt, Germany), according to the manufacturer’s instructions. Briefly, 50 μl Assay Diluent was added, and then standards or sample were added to the pre-coated microtiter plate followed by incubation for 2 h. Then, 100 μl MCP-1/IL-6 conjugate was immediately added. Then, 100 μl Substrate Solution was added followed by adding 100 μl Stop Solution. MCP-1 and IL-6 concentrations were calculated and normalized by total cell numbers.

### Adenovirus infection of 3T3-L1 adipocytes

Recombinant adenoviruses expressing XBP1 (Ad-XBP1) and GFP (Ad-GFP) were purchased from Obio Technology (Shanghai, China). For adenoviral infection, 3T3-L1 cells were infected with the adenovirus encoding XBP-1 at a multiplicity of infection of 20 for 48 h. Cells were then starved in medium containing 3% FBS for 16 h and then subjected to various treatments. Infection with the adenovirus encoding GFP was employed as a control.

### RNA interference and cell transfection

XBP-1 and control siRNAs were purchased from Qiagen (Valencia, CA). A 20 μM stock solution of XBP-1 siRNA or control scramble siRNA was prepared in siRNA dilution buffer. 3T3-L1 adipocytes were transfected in 6-well plates according to the manufacturer’s protocol. Briefly, for each transfection, high glucose DMEM containing 2% FBS without antibiotics was used as the transfection medium. Transfection medium (200 µl) containing 4 µl siRNA stock solution was incubated with 2 µl transfection reagent (Lipofectamine 2000, Invitrogen) for 45 min at room temperature. The siRNA-lipid complex which contained 1 ml transfection media was then added to each well. After 6 h of incubation, the transfection medium was replaced with growth medium containing 4% FBS without antibiotics for 16 h, which was then changed to normal growth medium for an additional 24 h before experiments. The protein level of XBP1 determined by western blot analysis is applied to assess the knockdown efficiency.

### Western blot analysis

Cells were lysed in ice-cold lysis buffer with a protease inhibitor cocktail (Santa Cruz Biotechnology, Santa Cruz, CA) for 30 min and then centrifuged at 13,300 rpm for 20 min. Thirty micrograms of protein were separated on an SDS-PAGE gel and then transferred. After blocking, the membrane was incubated overnight at 4 °C with a primary antibody including anti-phospho-NF-κB p65 (Ser536), anti-NF-κB, anti-phospho-IκB-α (Ser32), anti-IκB-α, anti-phospho-IKKα/β (Ser176/180) (Cell Signaling Technology, Boston, MA), anti-phospho-IRE1α (Abcam, Cambridge, MA), anti-IRE1α (Abcam, Cambridge, MA), or anti-β-actin (Cell Signaling Technology) antibodies. After incubation with an HRP-conjugated secondary antibody, signals were detected with a chemiluminescence western blotting detection solution using Bio Imaging System (Syngene, Frederick, MD).

### Statistical analysis

For quantification of the western blotting results, the intensities of bands were measured using image analysis software Multi Gauge, V3.0 (FUJIFILM, Tokyo, Japan). All experiments were performed with triplicates and repeated at least three times. Statistical analyses were performed using the Student’s *t* test, ANOVA, and Bonferroni’s multiple comparison test. *P* < 0.05 indicated significance.

## Results

### ER stress preconditioning attenuates the FFA-induced inflammatory response

**Fig. 1:**
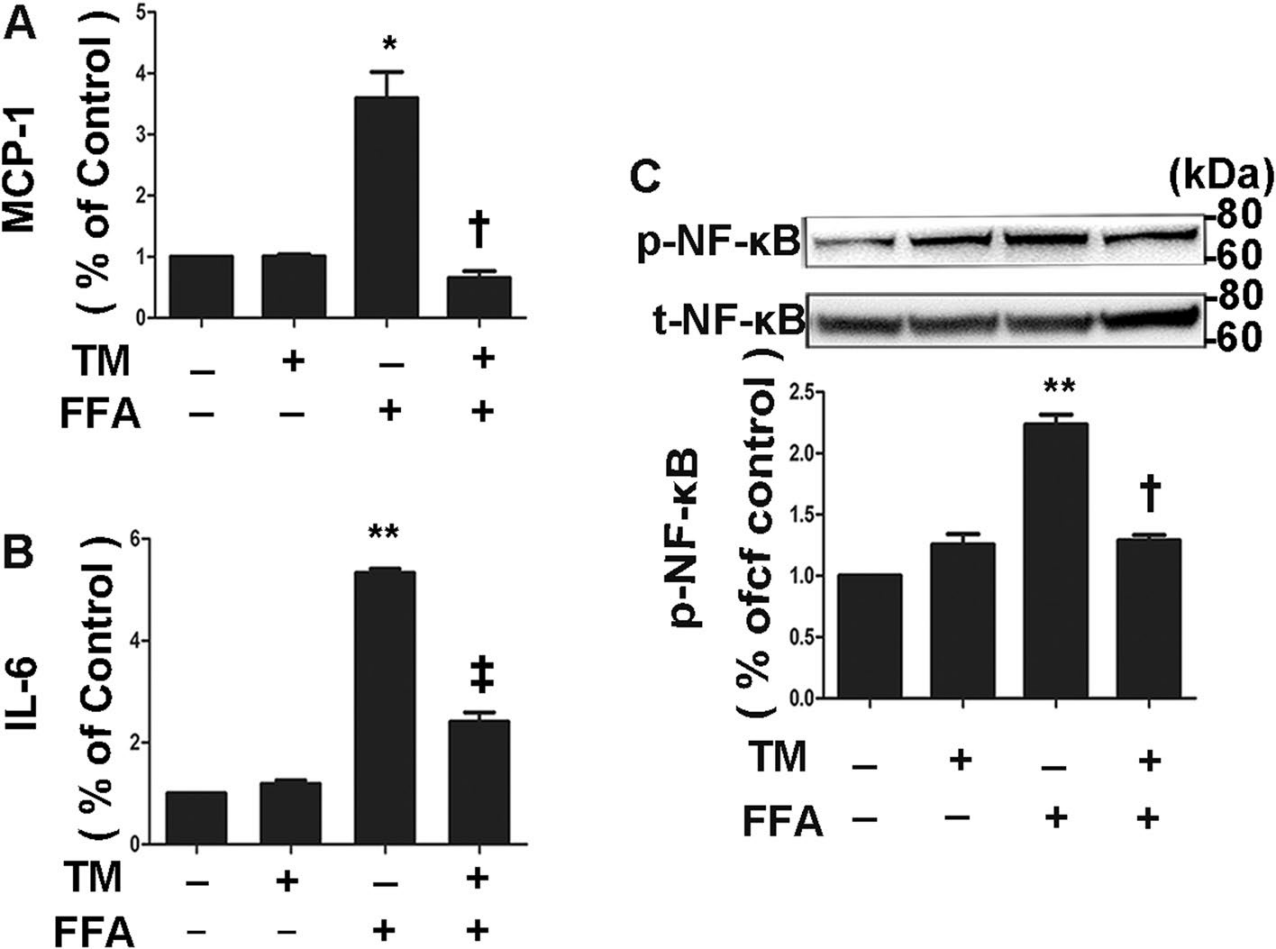
Inhibition of FFA-induced proinflammatory cytokines expression by ER stress preconditioning in 3T3-L1 adipocyte. **a**, **b** Expressions of MCP-1(**a**) and IL-6 (**b**) were determined by ELISA and normalized by cell number. 3T3-L1 adipocytes were preincubated with 0.5 µg/ml tunicamycin for 4 h followed by exposure to 0.5 mM FFA for 4 h. **c** Phosphorylation of NF-κB p65 subunit at Ser536 was determined by Western blot analysis and semi-quantified by densitometry. * for *P* < 0.05; ** for *P* < 0.01 versus control; ^†^ for *P* < 0.05, ^‡^, for *P* < 0.01 versus FFA. Results represent three independent experiments. Values were expressed as mean ± SD

Induction of inflammatory cytokine expression in 3T3-L1 adipocytes is a central step in the pathogenesis of adipocyte inflammation. We first determined whether ER stress preconditioning affected FFA-induced inflammatory cytokines expression in 3T3-L1 adipocytes. ER stress preconditioning was promoted by pretreatment of differentiated mature 3T3-L1 adipocytes with a very low dose of tunicamycin (0.5 μg/ml) for 4 h. Cells were then treated by FFAs (0.5 mM) for 4 h, followed by measurement of inflammatory protein levels. The results showed that MCP-1 and IL-6 secretion were upregulated by 10- and 8.5-fold, respectively, which were markedly inhibited by tunicamycin pretreatment (Fig. 1a and b).

NF-κB activation is a pivotal step in the overproduction of a variety of proinflammatory cytokines such as MCP-1 and IL-6. To testify whether preconditioning by ER stress affects activation of NF-κB in 3T3-L1 adipocytes, we analyzed phosphorylation of the p65 subunit of NF-κB at Ser536. Exposure of 3T3-L1 adipocytes to FFAs resulted in increased phosphorylation of the p65 subunit of NF-κB at Ser536 and the effect was largely inhibited by pretreatment with tunicamycin (Fig. 1c). This result indicates that ER stress preconditioning reduces the FFA-induced inflammatory response and NF-κB activation in 3T3-L1 adipocytes.

### ER stress preconditioning upregulates XBP1 expression in 3T3-L1 adipocytes

**Fig. 2:**
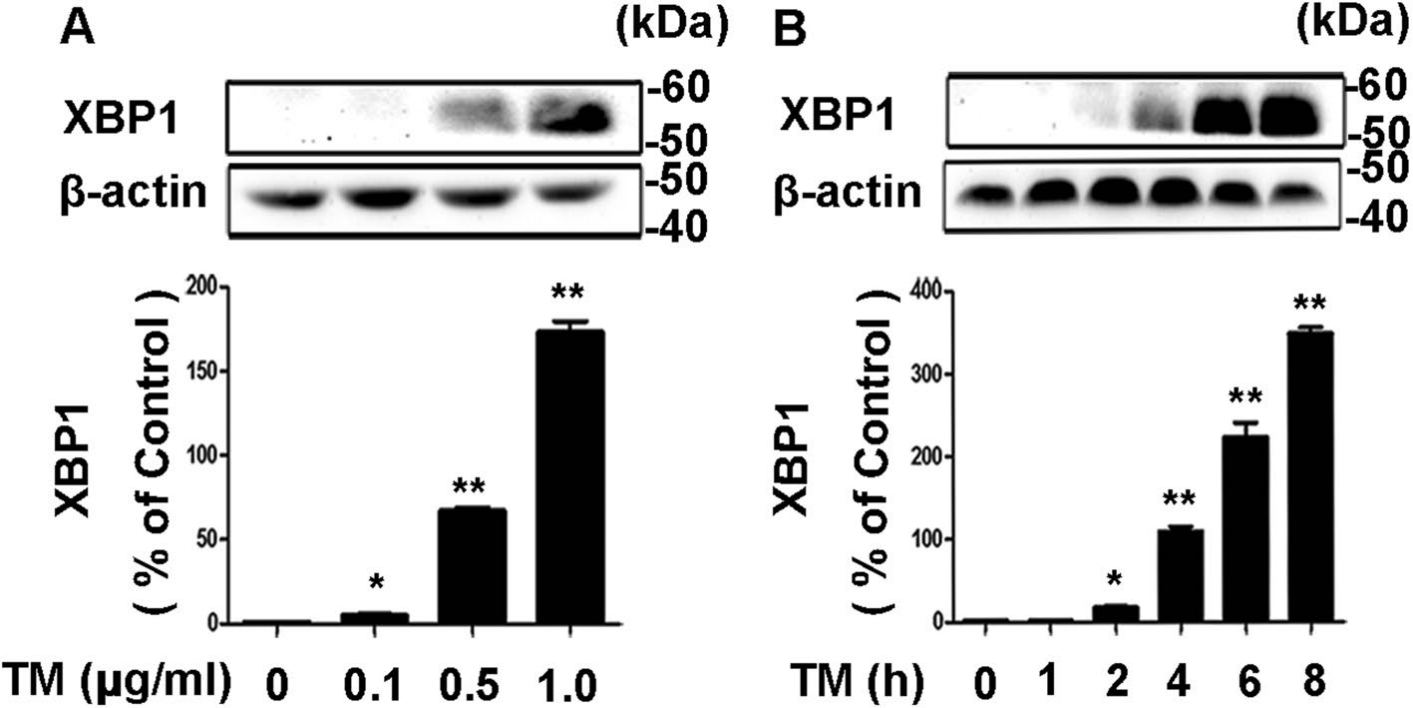
Tunicamycin treatment alone upregulates the expression levels of X-box-binding protein-1 (XBP1) in 3T3-L1 adipocytes in a dose-dependent and time-dependent manner. **a-b** The 3T3-L1 adipocytes were treated with tunicamycin (0.5 µg/ml) for the indicated dose (**a**) and the indicated durations (**b**), and a gene expression analysis of XBP1 was conducted. The results are expressed as the fold change. Results represent three independent experiments. Values were expressed as mean ± SD. * for *P* < 0.05; ** for *P* < 0.01 versus control

XBP1 activated by IRE1 is a key regulator of the adaptive UPR responding to ER stress. In addition, recent studies demonstrated that XBP1 is also an important player that mediated lipid metabolism [32, 33]. Then, we determined whether XBP1 is stimulated by ER stress preconditioning inducer tunicamycin. In our study, we found the expression of XBP1 is induced by tunicamycin at a dose-dependent manner (Fig. 2a). In addition, the protein level of XBP1 is upregulated from 4 h, peaked at 8 h, and declined at 12 h (Fig. 2b). These results indicate that expression of XBP1 is upregulated by ER stress preconditioning inducer tunicamycin in 3T3-L1 adipocytes.

### Silencing XBP1 blocks the protective effects of ER stress preconditioning on inflammation and NF-κB phosphorylation

**Fig. 3:**
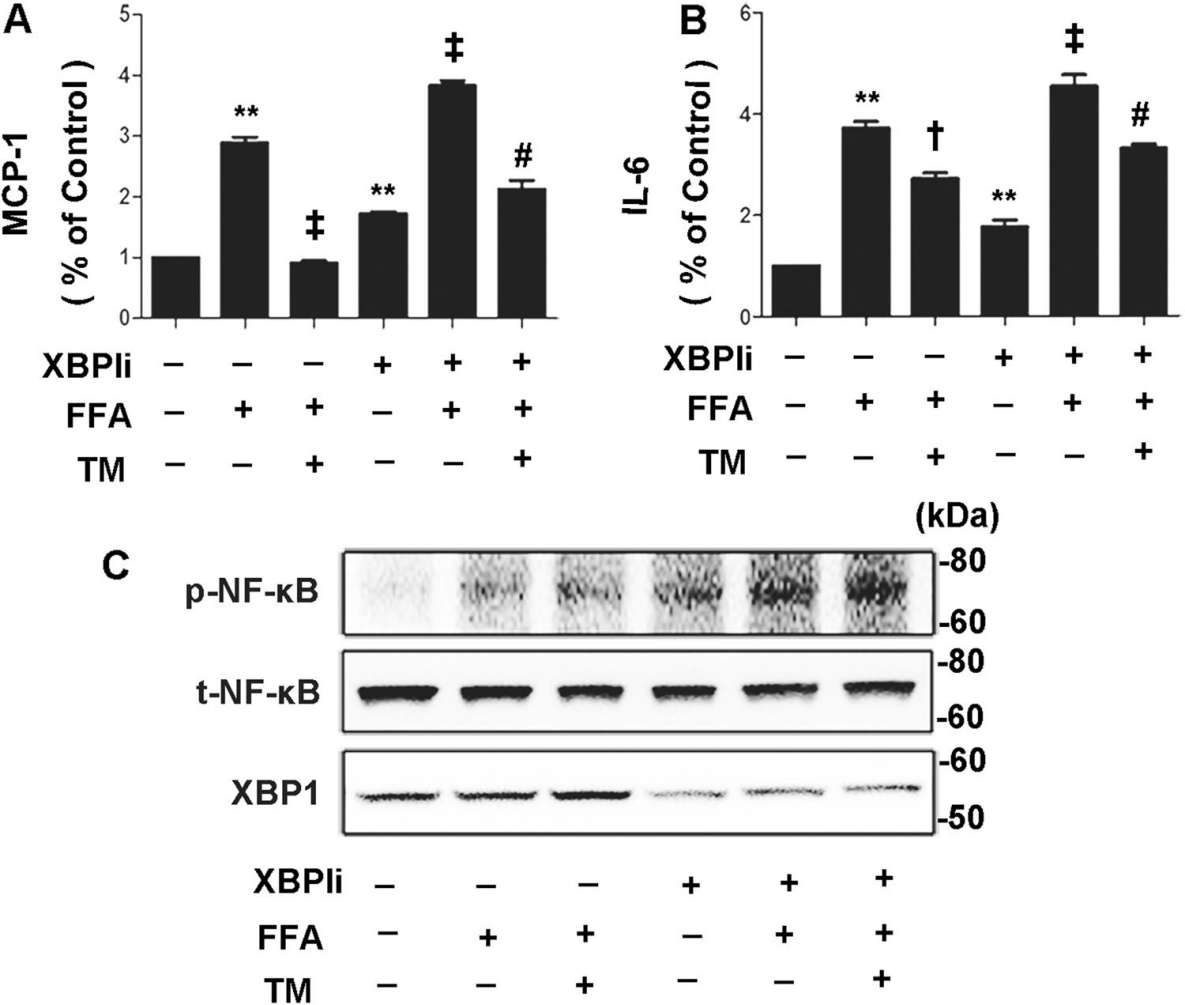
Genetic inhibition of XBP1 attenuated the protective effect of ER stress preconditioning in 3T3-L1 adipocyte. **a-b** Expressions of MCP-1 (**a**) and IL-6 (**b**) were determined by ELISA and normalized by cell number. **c** Phosphorylation of NF-κB p65 subunit at Ser536 was determined by Western blot analysis. 3T3-L1 adipocytes were transfected with XBP1siRNA for 48 h followed by exposure to 0.5 mM FFA for 4 h. * for *P* < 0.05 versus Ctrl. **P < 0.01 versus control; ^‡^*P* < 0.01 versus FFA; ^#^*P* < 0.05 versus FFA + tunicamycin

**Fig. 4:**
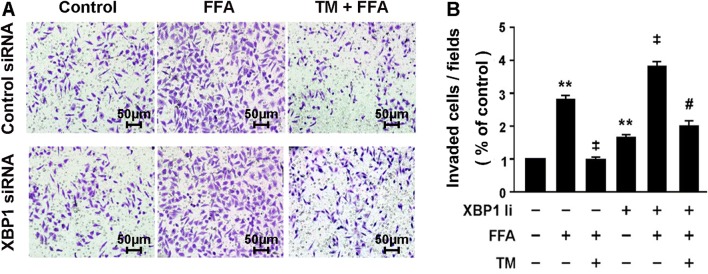
Genetic inhibition of XBP1 suppressed the protective effect of ER stress preconditioning on macrophage motility. **a** Control siRNA (**A**); Control siRNA +FFA (B); Control siRNA + TM + FFA (C); D: XBP1siRNA (D); E: XBP1siRNA + FFA (E); XBP1siRNA + TM + FFA (F). Scale bar, 50 μm. **b** Quantification of migrated cells was counted as the mean number of cells per field of view. RAW 264.7 macrophages were treated with DMEM contained 2% FBS or 3T3-L1 conditioned medium for 4 h. Migrated RAW264.7 macrophages were quantified and expressed as the mean number of cells per field of view. A: Migrated RAW264.7 macrophages were stained with hematoxylin. Representative results were from three independent experiments. * for *P* < 0.05 versus Ctrl. **P < 0.01 versus control; ^‡^*P* < 0.01 versus FFA; ^#^*P* < 0.05 versus FFA + tunicamycin

We next determined the role of XBP1 in the anti-inflammatory effects stimulated by FFA in 3T3-L1 adipocytes. As shown in Fig. 3, pretreatment with XBP1siRNA significantly restored the secretion of MCP-1 (*P* < 0.05; Fig. 3a) and IL-6 (*P* < 0.05; Fig. 3b) in FFA-treated 3T3-L1 adipocytes with ER stress preconditioning. Moreover, XBP1siRNA blocked the protective effect of ER stress preconditioning on RAW264.7 macrophages migration (Fig. 4a, b) and NF-κB phosphorylation (Fig. 3c). These results collectively suggest that XBP1 plays a beneficial role in ER stress preconditioning in inhibiting the inflammatory response of 3T3-L1 adipocytes.

### XBP1 ameliorates FFA-induced inflammation and suppresses NF-κB phosphorylation

**Fig. 5:**
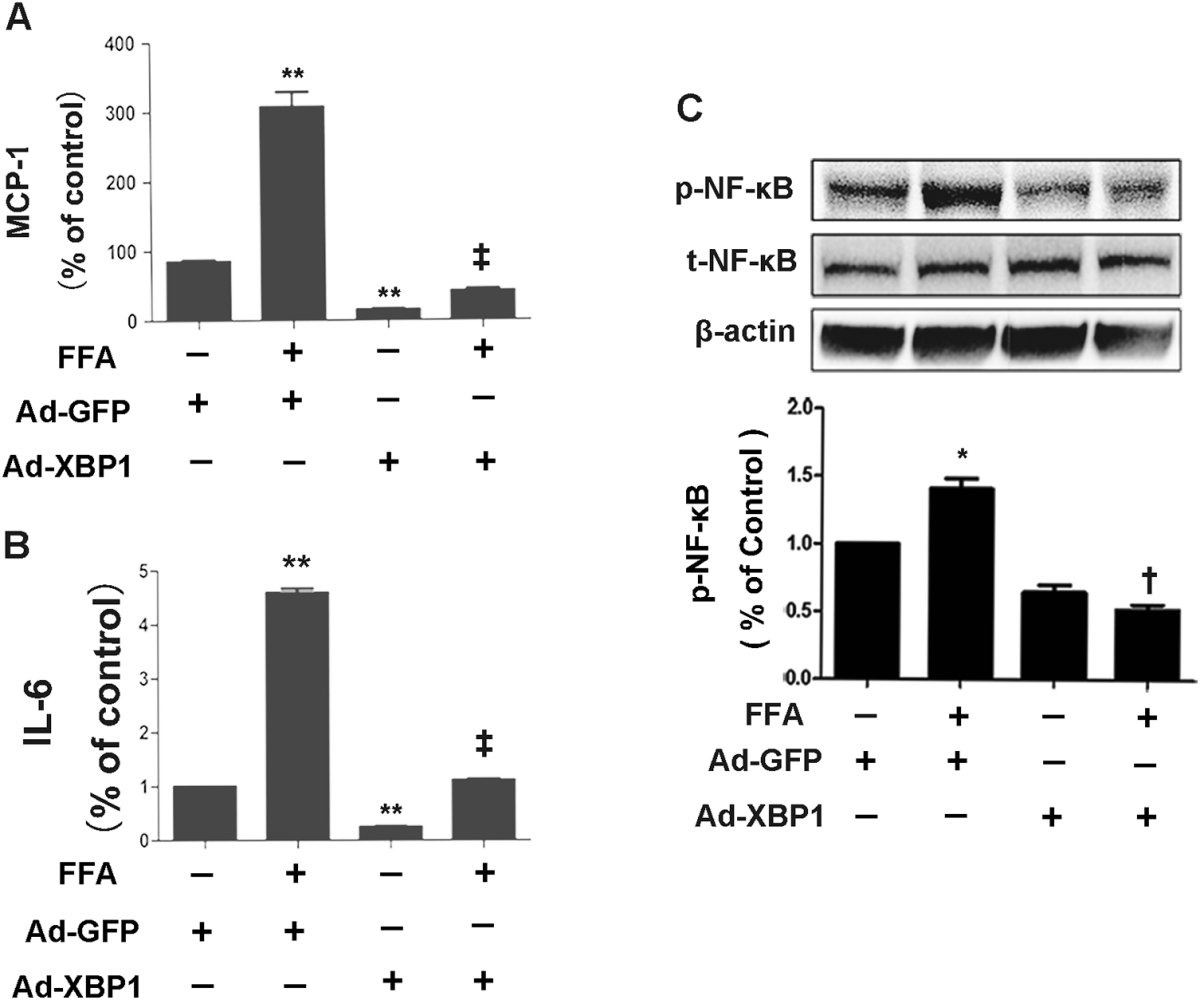
Overexpression of XBP1 inhibited FFA-induced proinflammatory cytokines expression via suppression of NF-κB activation in 3T3-L1 adipocytes. **a**, **b** Expressions of MCP-1 (**a**) and IL-6 (**b**) were determined by ELISA and normalized by cell number. **c** Phosphorylation NF-κB p65 (Ser536) was determined in 3T3-L1 adipocytes after treatment with FFA for 4 h. Total NF-κB was also determined by Western blot analysis and semi-quantified by densitometry. Overexpression of XBP1 was achieved by infection of 3T3-L1 adipocytes with adenovirus encoding with XBP1 at a multiplicity of infection of 20 for 48 h. Adenovirus encoding GFP (Ad-GFP) was used as control. After infection with Ad-GFP and Ad-XBP1, 3T3-L1 adipocytes were treated with FFA for 4 h. Representative results were from three independent experiments. *P < 0.05; **P < 0.01 versus Ad-GFP; ^†^*P* < 0.05; ^‡^*P* < 0.01 versus Ad-GFP + FFA

To further verify whether XBP1 directly regulated the inflammatory response of FFA-treated 3T3-L1 adipocytes, we overexpressed XBP1 in 3T3-L1 adipocytes by infection of cells with adenovirus encoding mouse XBP1 (Ad-XBP1). Adenovirus encoding GFP (Ad-GFP) was used as control. The overexpression of XBP1 in the 3T3-L1 adipocytes was confirmed by western blot analysis (data not shown). Inflammatory molecule secretion was determined by ELISAs and NF-κB phosphorylation was determined by western blot analysis. The results showed that FFA-induced robust increases of MCP-1 and IL-6 secretion (Fig. 5a, b), and NF-κB phosphorylation in Ad-GFP-treated cells, but not in Ad-XBP1-treated cells (Fig. 5c).

### XBP1 inhibits IKK activation and suppresses IRE phosphorylation

**Fig. 6:**
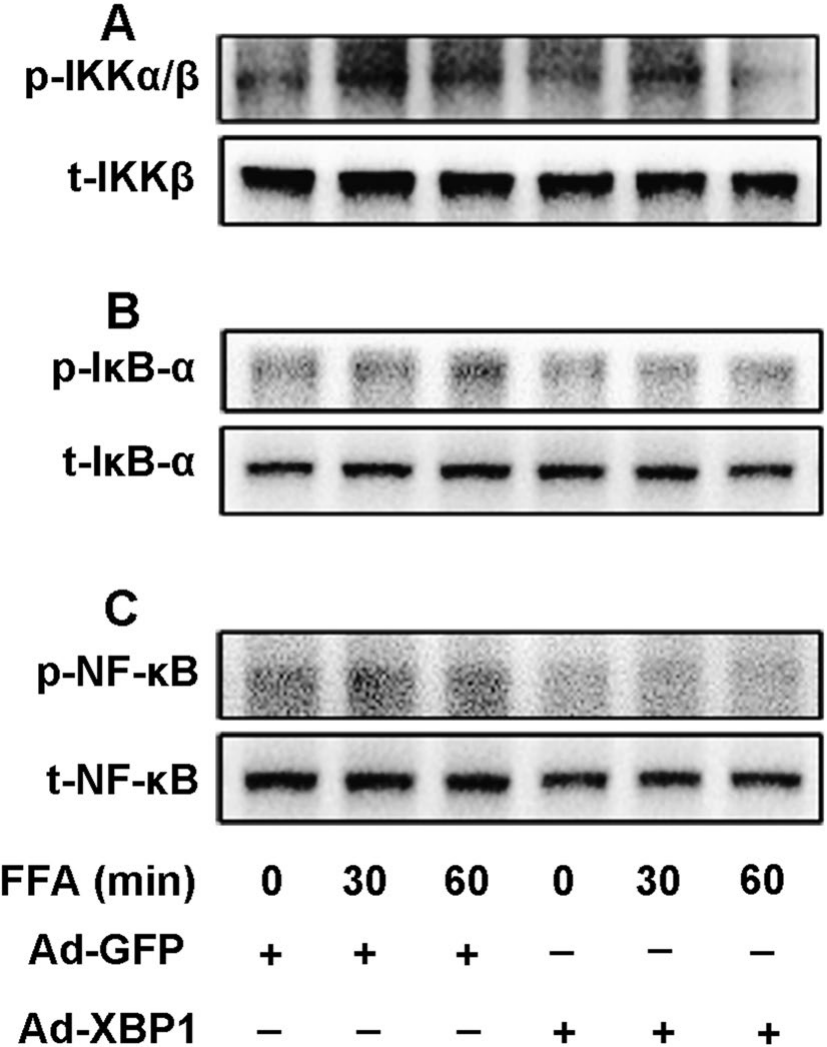
Overexpression of XBP1 inhibited FFA-induced IKK activation. **a**–**c** Phosphorylation of IKKα/β at Ser176/180 (**a**), IκB-α at Ser32 (**b**), and NF-κB at Ser536 (**c**) were detected by Western blot analysis. 3T3-L1 adipocytes were infected with adenovirus encoding with XBP1 (Ad-XBP1) at a multiplicity of infection of 20 for 48 h. Adenovirus encoding GFP (Ad-GFP) was used as control. After infection with Ad-GFP and Ad-XBP1, 3T3-L1 adipocytes were treated with 0.5 mM FFA for up to 60 min. Total IKKα, IKKβ, IκB-α, and NF-κB were also determined. Representative results were from three independent experiments

**Fig. 7:**
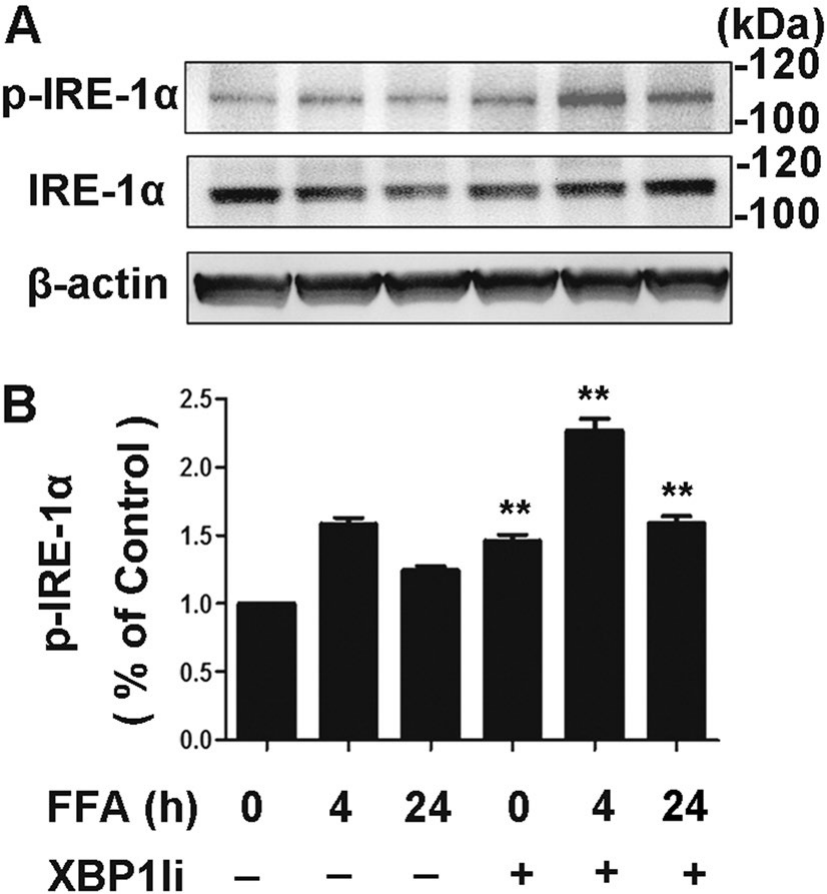
XBP1 blocked FFA-induced IKK activation through inhibition of IRE phosphorylation. **a**, **b** Expressions of p-IRE-1α (**a**), total phospho-IRE-1α (**b**), and β-actin were determined by Western blot analysis and semi-quantified by densitometry. 3T3-L1 adipocytes were infected with XBP-1siRNA or control siRNA for 48 h, followed by treatment with 0.5 mM FFA for 4 h and 24 h. Representative results were from three independent experiments. **P < 0.01 versus control or control siRNA

IKK activation is an essential step in inflammatory pathways that lead to NF-κB activation. IKK upregulates the phosphorylation of the protein IκB that lead to the nuclear translocation of NF-κB [39, 40] and activates inflammatory response. To investigate whether NF-κB activation is regulated by XBP1 through IKK, we determined the effect of XBP1 on FFA-induced phosphorylation of IKK by overexpression of XBP1 via adenovirus infection. As shown in Fig. 6, treatment with FFAs induced a time-dependent increase in the phosphorylation of IκB-α, NF-κB p65, and IKKα/β which is markedly reversed by Ad-XBP1 transfection in 3T3-L1 adipocytes as determined by western blot analysis (Fig. 6a–c), while the total protein expression levels of IKK-α/β, IκB-α, and NF-κB p65 remained unchanged. These results confirmed the reduction of the inflammatory effect and activation of the NF-κB pathway by FFAs in 3T3-L1 adipocytes, which may in part contribute to XBP1. Activation of XBP1 requires splicing by IRE1α. To further assess the mechanisms of how XBP1 regulates the IKK pathway, we examined the phosphorylation of IRE1α in FFA-stimulated cells. Results of western blot analysis showed that transfection with XBP1siRNA led to upregulated phosphorylation of IRE1 in FFA-treated 3T3-L1 adipocytes, but the total level of IRE1α had not been changed (Fig. 7).

**Fig. 8:**
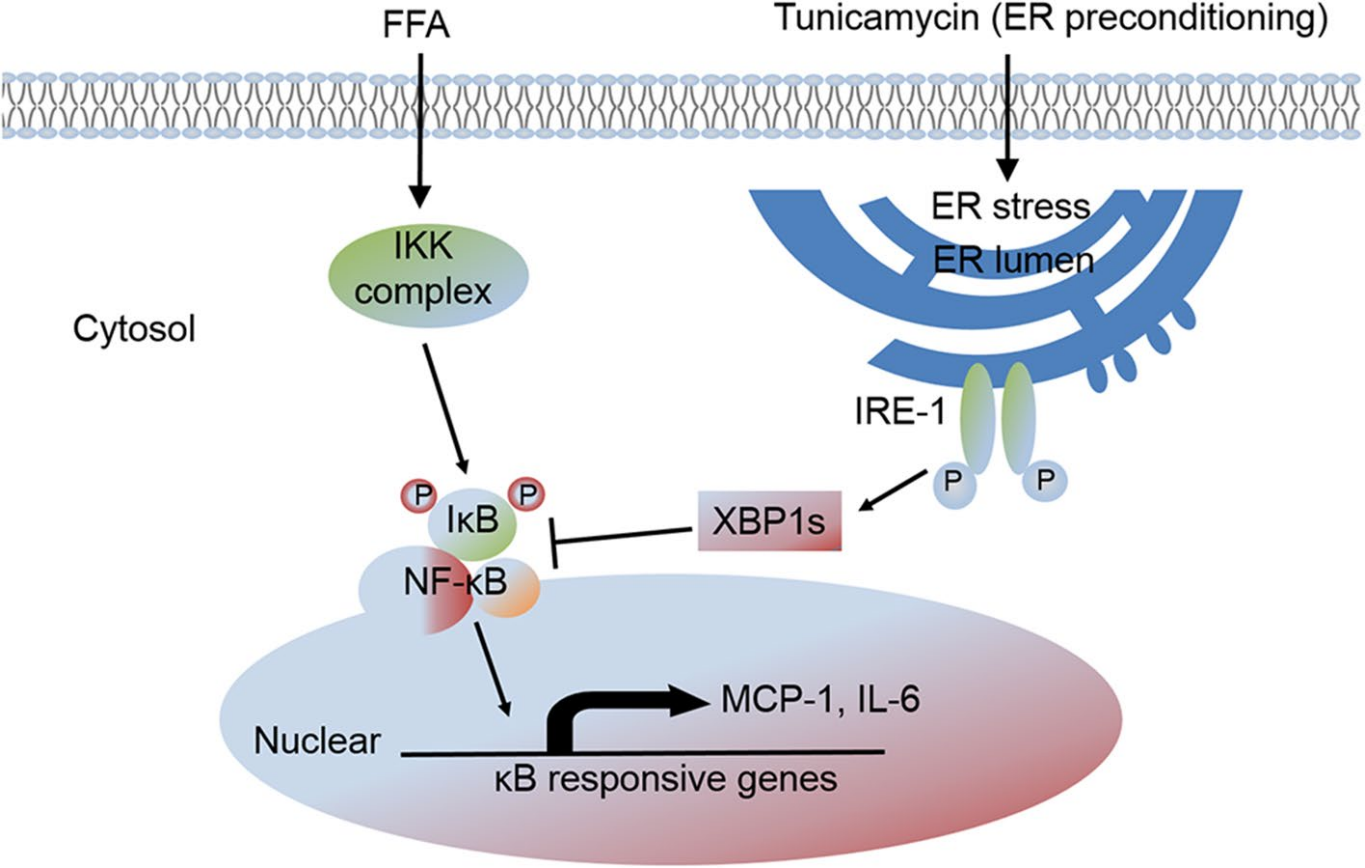
Role of XBP1 in Tunicamycin-induced ER stress preconditioning in FFA-stimulated inflammatory gene expression in 3T3-L1 adipocytes. ER stress preconditioning via tunicamycin elevates XBP1s level, which inhibited FFA-stimulated NF-κB pathway and thus suppressed NF-κB activated inflammatory genes in 3T3-L1 adipocytes

In conclusion, our data indicate that ER stress preconditioning can protect 3T3-L1 adipocytes against FFA-induced inflammatory response by the induction of XBP1. The upregulation of XBP1 led to the inhibition of IRE1/IKK/NF-κB pathway which in turn upregulates inflammatory gene expression, leading to adipocyte inflammation and obesity (Fig. 8).

## Discussion

Obesity is strongly associated with cardiovascular disorders and diabetes. Recent studies have shown that excess energy intake and endogenous lipolysis of saturated fatty acids might increase the production of proinflammatory cytokines, including IL-6 and MCP-1 as well as activation of NF-κB signaling pathway, which partly contributed to adipocyte inflammation and obesity [34–36]. Inhibition of the NF-κB signaling pathway in 3T3-L1 adipocytes not only prevents adipocyte inflammation, but also ameliorates adipocyte dysfunction and increases insulin sensitivity [37, 38]. The NF-κB pathway is vital for the inflammatory response, which has been proven in several types of mature cells and various cellular signals. P-IKKα/β, p-IκBα, and p-p65 are the phosphorylated forms of three important proteins in the NF-κB pathway [35, 39, 40]. Our previous study demonstrated that 0.5 mM FFAs upregulate phosphorylation of the NF-κB p65 subunit at Ser536, leading to overproduction of IL-6 and MCP-1 in 3T3-L1 adipocytes. In the present study, we demonstrated that preconditioning with ER stress attenuated FFA-induced expression of inflammatory cytokines and NF-κB activation in 3T3-L1 adipocytes. In addition, the expressions of p-IKKα/β, p-IκBα, and p-p65 in 3T3-L1 adipocytes were suppressed by ER stress preconditioning in time-dependent manners. Our results also indicate that activation of XBP1 is essential for the protective effects of ER stress preconditioning through mediation of the IRE1/IKK/NF-κB pathway. This study for the first time elucidated a beneficial effect and involved mechanisms of ER stress preconditioning on regulation of adipocyte inflammation, which can help to elucidate the specific molecular mechanism of FFA-induced adipocyte inflammation and will provide theoretical basis for the treatment of obesity and metabolic disorders.

There is a close link between ER stress and the inflammatory response. In our previous study, we found that induction of ER stress contributes to retinal inflammation in diabetic retinopathy [41]. Jiao et al. [34] demonstrated that ER stress is a key mediator of FFA-induced inflammation in adipocytes together with PKR-like eukaryotic initiation factor 2α kinase, one of the three major ER stress sensor proteins. Accumulating evidence indicates a protective effect of ER stress preconditioning against the cytokine-induced inflammatory response. Yu et al. [42] demonstrated that preconditioning by ER stress suppresses acrylonitrile-induced cytotoxicity in primary rat astrocytes. Zhang et al. [25] showed that preconditioning with ER stress ameliorates inflammation in retinal endothelial cells by suppressing NF-κB-mediated adhesion molecule expression. Pretreatment with sub-nephritogenic doses of ER stress inducers tunicamycin or thapsigargin in rats mitigates mesangioproliferative glomerulonephritis [21]. These studies suggest a protective role of signaling pathways activated by ER stress against inflammatory conditions. In the present study, we showed that FFA-induced inflammatory cytokine production was significantly suppressed in ER stress-pretreated 3T3-L1 adipocytes, suggesting a protective role of ER stress preconditioning, which is in line with previous investigations.

XBP1s is an important gene that turns on transcription of a subset of ER chaperones genes in response to stress. Previous studies have reported a pivotal role of XBP1 in the development of several diseases such as Alzheimer’s Disease [43], diabetic retinopathy [44], neurodegeneration [45], insulin resistance [46], and obesity [47]. Sha et al. [48] demonstrated that XBP1s improves glucose tolerance and insulin sensitivity in both lean and obese (ob/ob) mice by promoting adiponectin multimerization. Iwakoshi et al. [49] reported that XBP1 is required for plasma cell differentiation and the UPR. However, there are still some conflicting studies concerning the role of XBP1 during cell development. Claudio et al. [50] reported that XBP-1-deficient mice are more resistant to developing disease, which correlated with increased levels of autophagy in motoneurons. Our data indicated that overexpression of XBP1 suppressed FFA-induced secretion of inflammatory cytokines. Importantly, the present study demonstrated that pretreatment of 3T3-L1 adipocytes with the ER stress inducer tunicamycin upregulated XBP1, but not proinflammatory cytokines.

Previous groups have been reported that co-culture of 3T3-L1 adipocyte and macrophages activates the proinflammatory response and in turn upregulates the motility of macrophages [51]. In our study, we demonstrated that co-culture of 3T3-L1 adipocyte and macrophages increases the macrophages migration which is in line with the result of previous studies. Furthermore, we demonstrated that XBP1 siRNA blocks the inhibitory effect of ER stress preconditioning on FFA-induced macrophages migration which in turn augments the adipocyte inflammation. Little literatures have reported the effect of XBP1s on macrophage migration and adipocyte inflammation. Zhang and associates demonstrated that quinotrierixin, a specific inhibitor of XBP1 splicing, could increase the leukocyte adhesion to endothelial cells in the dose-dependent manner [25]. In addition, Ozcan et al. [52] showed that increased XBP1s activity leads to reduced ER stress, and improved insulin sensitivity and glucose homeostasis. Moreover, studies show that XBP1 deletion triggers feedback activation of its upstream enzyme, IRE1α [53, 54]. Sha et al. [55] reported that IRE1α could promote glucose tolerance and insulin sensitivity through activated XBP1s in both starved and obese mice. In our study, we found that XBP1 negatively regulated IRE1 activation, a critical step for FFA-induced IKK and NF-κB activation, in both untreated and FFA-treated cells, which is consistent with a study by Zhang et al. [26]. Our findings are consistent with these results and highlight possible protective roles of anti-inflammatory and anti‑obesity effects of XBP1s. However, the mechanisms by which XBP1 activates IRE1α need to be investigated further.

In conclusion, our study demonstrates that preconditioning with ER stress attenuates FFA-induced inflammation in 3T3-L1 adipocytes. XBP1 plays an essential role in the protective effects of ER stress preconditioning and is required to inhibit FFA-induced inflammation mediated by the IRE1/IKK/NF-κB pathway. To our knowledge and as summarized in Fig. 8, this is the first study providing results of the protective role of ER stress preconditioning in FFA-induced adipocyte inflammation to exert the anti-obesity effect. Identifying small molecules that enhance XBP1-mediated protective UPR signaling may provide a novel therapeutic strategy against adipocyte inflammation and obesity. Our study lacks in vivo data, which is a limitation. Thus, researches into the use of ob/ob mice or C57BL6/J mice with high-fat-diet-induced obesity as a target for metabolic diseases hold much promise.
